# Individualized response to semantic versus phonological aphasia therapies in stroke

**DOI:** 10.1093/braincomms/fcab174

**Published:** 2021-08-05

**Authors:** Sigfus Kristinsson, Alexandra Basilakos, Jordan Elm, Leigh Ann Spell, Leonardo Bonilha, Chris Rorden, Dirk B den Ouden, Christy Cassarly, Souvik Sen, Argye Hillis, Gregory Hickok, Julius Fridriksson

**Affiliations:** 1 Department of Communication Sciences and Disorders, University of South Carolina, Columbia, SC 29208, USA; 2 Center for the Study of Aphasia Recovery, University of South Carolina, Columbia, SC 29208, USA; 3 Department of Public Health Sciences, Medical University of South Carolina, Charleston, SC 29425, USA; 4 Department of Neurology, Medical University of South Carolina, Charleston, SC 29425, USA; 5 Department of Psychology, University of South Carolina, Columbia, SC 29208, USA; 6 Department of Neurology, University of South Carolina, Columbia, SC 29208, USA; 7 Department of Neurology and Physical Medicine & Rehabilitation, Johns Hopkins School of Medicine, Baltimore, MD 21218, USA; 8 Department of Cognitive Science, Johns Hopkins University, Baltimore, MD 21218, USA; 9 Department of Cognitive Sciences and Language Science, University of California, Irvine, CA 92697, USA

**Keywords:** stroke, aphasia, aphasia therapy, phonological therapy, semantic therapy

## Abstract

Attempts to personalize aphasia treatment to the extent where it is possible to reliably predict individual response to a particular treatment have yielded inconclusive results. The current study aimed to (i) compare the effects of phonologically versus semantically focussed naming treatment and (ii) examine biographical and neuropsychological baseline factors predictive of response to each treatment. One hundred and four individuals with chronic post-stroke aphasia underwent 3 weeks of phonologically focussed treatment and 3 weeks of semantically focussed treatment in an unblinded cross-over design. A linear mixed-effects model was used to compare the effects of treatment type on proportional change in correct naming across groups. Correlational analysis and stepwise regression models were used to examine biographical and neuropsychological predictors of response to phonological and semantic treatment across all participants. Last, chi-square tests were used to explore the association between treatment response and phonological and semantic deficit profiles. Semantically focussed treatment was found to be more effective at the group-level, independently of treatment order (*P* = 0.041). Overall, milder speech and language impairment predicted good response to semantic treatment (*r* range: 0.256–0.373) across neuropsychological tasks. The Western Aphasia Battery-Revised Spontaneous Speech score emerged as the strongest predictor of semantic treatment response (*R*^2^ = 0.188). Severity of stroke symptoms emerged as the strongest predictor of phonological treatment response (*R*^2^ = 0.103). Participants who showed a good response to semantic treatment were more likely to present with fluent speech compared to poor responders (*P* = 0.005), whereas participants who showed a good response to phonological treatment were more likely to present with apraxia of speech (*P* = 0.020). These results suggest that semantic treatment may be more beneficial to the improvement of naming performance in aphasia than phonological treatment, at the group-level. In terms of personalized predictors, participants with relatively mild impairments and fluent speech responded better to semantic treatment, while phonological treatment benefitted participants with more severe impairments and apraxia of speech.

## Teaser

Kristinsson et al. found superior effects of semantic versus phonological therapy in a group of 104 participants with chronic aphasia. Personalized predictors of semantic therapy response included mild language impairment and fluent speech, whereas more severe language impairment and presence of apraxia of speech predicted response to phonological therapy.

## Graphical Abstract

**Graphical Abstract:**
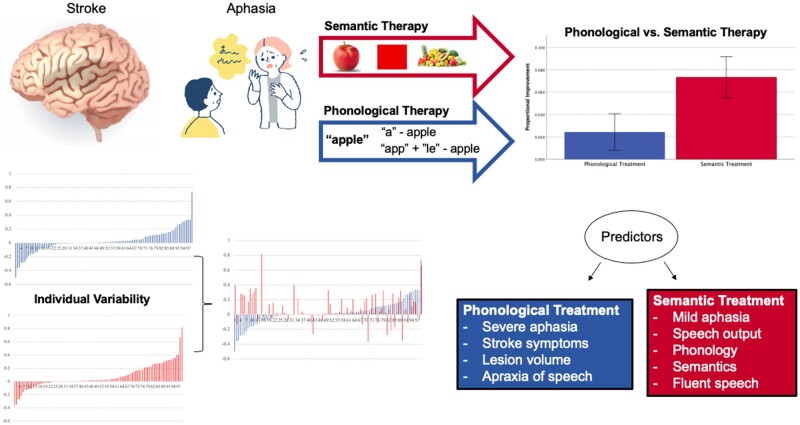

## Introduction

Aphasia is a debilitating language disorder most frequently resulting from a left-hemisphere stroke. The degree to which persons with aphasia (PWA) experience an impairment in language comprehension and expression can range from mild difficulties to a complete loss of language.^1^ Aphasia has been shown to have detrimental effects on activities of daily living, including return to work, social relations, and quality of life.^1–4^ Therefore, it serves as a clinically important goal to study treatment efficacy in aphasia and, specifically, to advance personalized treatment aiming to maximize each individual’s potential recovery.

Aphasia treatment is beneficial for improving functional communication and language outcomes at the group-level.^5–8^ Furthermore, the persistent notion that PWA experience a plateau in recovery after the first year post-stroke^9–11^ has been challenged with compelling evidence suggesting that language recovery is dynamic^12–16^ and, crucially, that behavioural treatment is a driving factor in improving language function in chronic aphasia.^8^^,^^15^^,^^17–19^

While several studies have aimed to identify individual predictors of treatment response in aphasia based on biographical,^9^^,^^15^^,^^20^ neuropsychological^21–26^ and neuroimaging variables,^27–33^ the findings to date do not enable clinicians and researchers to reliably personalize treatment for any single individual with aphasia to the extent where they can confidently predict potential language gains based on baseline assessments. To this end, there is furthermore a notable lack of conclusive evidence regarding the intuitively important question of who benefits from what type of restorative treatment paradigm^19^^,^^28^^,^^34–42^—a question that is crucial for clinicians treating aphasia. This issue was illustrated in a recent review of advances in clinical trials of aphasia treatments in the past 5 years,^43^ wherein the authors concluded that studies focussing on personalized treatment were substantially underrepresented within the field of aphasia treatment research.

Anomia, or the inability to retrieve a target word from the mental lexicon, may be a particularly promising target to study personalized aphasia treatment as it is commonly considered a hallmark symptom that affects virtually all PWA.^44^ Highly influential models of word retrieval specify that successful naming requires processing at the semantic and phonological level.^45–48^ Anomia, therefore, may be caused by impaired processing in one or both of these processing levels, or in the connection between levels. In line with this, treatment approaches to improve naming typically employ a semantically or phonologically based focus.^19^^,^^41^^,^^49^^,^^50^ Semantic approaches aim to strengthen semantic representations through various task paradigms,^51^ including generation of semantic features of a target word and semantic feature verifications.^52–55^ Phonological approaches, on the other hand, aim to strengthen representations at the word-form level or connections from the semantic system to the word form.^51^ Similar to semantic treatment, phonological treatment tasks may include leveraging phonological information via cueing hierarchies and phonological feature generation.^56–58^ There is, indeed, ample evidence suggesting that both treatment paradigms do elicit lasting gains in naming performance in aphasia.^19^^,^^34^^,^^37^^,^^38^^,^^41^^,^^49^^,^^50^^,^^55^^,^^58–70^

Although both semantically and phonologically based approaches are effective, the precise mechanism by which treatment gains are elicited with each treatment has long been debated.^38^^,^^41^^,^^71^ Nickels^41^ considers impaired word retrieval a symptom that can have its cause in a number of different underlying impairments. Based on this rationale, Nickels argues that there is no reason to assume that any one treatment paradigm induces language gains for all individuals with aphasia. Following this line of thought, one hypothesis posits that each different level of breakdown in word production will be best remediated by a different type of treatment.^39^^,^^72^^,^^73^ Howard,^71^ on the contrary, argues that the difference between semantically and phonologically based treatments is more apparent than real.^71^ Specifically, Howard considers both treatment types to have their effect in the same way—by strengthening the mapping between semantic and phonological word forms when both are simultaneously active.^74^ These conflicting views highlight the need for a detailed investigation of who benefits from each treatment type.

Despite the considerable research effort that has been undertaken to inform this question, the evidence regarding which treatment type to implement with a specific patient remains inconclusive. Several studies have compared the effects of phonological and semantic treatment across or within individuals, and have examined predictors of treatment response. Briefly, some evidence suggests that semantic treatment may lead to greater generalization to untrained items^19^^,^^37^^,^^38^^,^^69^^,^^75^, but evidence showing favourable outcome after phonological treatment also exists.^73^^,^^74^^,^^76–78^ Furthermore, several studies have suggested that using both approaches improves naming abilities in the same individuals^35^^,^^36^^,^^38^^,^^75^^,^^79^^,^^80^, and others have similarly failed to find a consistent relationship between participant deficit profiles and success with particular treatment types^39^^,^^41^^,^^42^^,^^70^ (see ref. 79 for contrasting findings).

Taken together, prior studies hardly enable clinicians to accurately match a patient with a treatment type that may maximize potential recovery. In an attempt to shed further light on the conflicting findings, Gilmore et al.^81^ examined the predictive value of cognitive skills for naming outcome after semantic feature analysis in 99 PWA. The findings revealed that 54% of variance in treated recovery could be explained by measures of executive function, verbal and visual short-term memory. These results may be taken to suggest that factors beyond impaired language processes need to be considered when investigating response to a given treatment type. To our knowledge, other studies have not explicitly aimed to predict treatment response to phonological and semantic treatment based on an extensive set of neuropsychological baseline tests, incorporating measures of language function and cognitive abilities.

The current study seeks to address the problems described above. Specifically, we addressed the following aims: (i) Do phonological and semantic treatments yield comparable improvement in naming within a single participant cohort? and (ii) What biographical and neuropsychological baseline factors predict response to phonological and semantic treatment? As such, this study offers the novelty of (i) providing the largest trial to date comparing the effects of phonological and semantic treatments in aphasia and (ii) identifying personalized predictors of response to each treatment type separately.

## Method

Data for the current study were obtained under the POLAR (**P**redicting **O**utcome of **La**nguage **R**ehabilitation in Aphasia) protocol. A detailed discussion of the aims of the POLAR trial is provided in Basilakos et al.^82^

### Participants

Participants were recruited through the University of South Carolina (USC) and Medical University of South Carolina (MUSC) and all study procedures have been approved by Institutional Review Boards at both universities. At the time of database freeze (3 December 2021), a total of 104 participants had completed all study procedures. Participants had chronic aphasia due to left-hemisphere stroke (≥12 months post-onset), as diagnosed by the Western Aphasia Battery-Revised (WAB-R)^83^; were between 21 and 80 years of age; speakers of English as their primary language for ≥20 years; willing and able to provide informed consent; and were able to undergo MRI scanning. Individuals with multiple strokes were recruited, as long as all lesions were confined to the left supratentorial hemisphere. Exclusion criteria were severely limited verbal output (WAB-R Spontaneous Speech score of 0–1), severely impaired auditory comprehension (WAB-R Auditory Comprehension score of 0–1) and bilateral stroke. Table 1 presents participants’ characteristics.

**Table 1:** Participants' characteristics across treatment groups (phonological treatment first, *n* = 50; semantic treatment first, *n* = 49)

Measure	Treatment group		Two-tailed *P*-valuea
	Phonological first (*n* = 50)	Semantic first (*n* = 49)	
F/M^b^	19/31	22/27	0.485
Age	61.8 years (SD = 11.4)	60.0 years (SD = 10.4)	0.454
Education	15.4 years (SD = 2.4)	15.4 years (SD = 2.2)	0.945
MPO	49.4 months (SD = 52.8)	43.9 months (SD = 49.6)	0.596
Lesion volume	1,315 cc (SD = 846)	1,182 cc (SD = 952)	0.481
WAB-AQ	58.6 (SD = 22.2)	59.4 (SD = 22.6)	0.858
PNT baseline	77.2 (SD = 61.2)	77.0 (SD = 58.8)	0.988
NIHSS	6.0 (SD = 3.6)	6.4 (SD = 3.7)	0.633

F/M, female/male; MPO, months post-onset; NIHSS, National Institute of Health Stroke Scale; PNT baseline, Philadelphia Naming Test baseline score; WAB-AQ, Western Aphasia Battery Aphasia Quotient.

a Independent samples *t*-tests were used for all comparisons, unless otherwise denoted.

b *P*-value based on chi-square statistic.

Of note, the last 11 participants entered the study after the outbreak of the COVID-19 pandemic; data collection and treatment were carried out virtually for these participants. Study parameters were held as constant as logically possible and primary outcome measures remained the same, but a less extensive baseline assessment protocol was utilized to adhere to assessment reliability guidelines and participants’ needs. The updated COVID-19 study protocol was approved by the Institutional Review Boards at both USC and MUCS. Potential implications of these changes are discussed below as applicable.

### Study timeline

**Figure 1:**
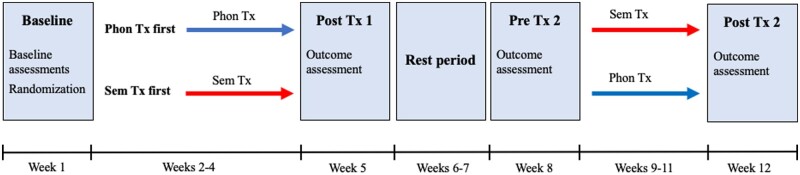
**Study timeline.** Phon Tx first = phonological treatment followed by semantic treatment; Sem Tx first = semantic treatment followed by phonological treatment; Phon Tx = phonologically focussed treatment; Sem Tx = semantically focussed treatment; Post Tx 1 = post-treatment phase 1; Post Tx 2 = post-treatment phase 2.

Although the POLAR trial follows participants through a 1- and 6-month post-treatment follow-up, the current analyses consider treatment-induced change immediately following two rounds of therapy, i.e. the first 12 weeks of the study. During week 1, baseline testing was conducted, and participants were randomized to treatment groups. The first treatment phase covered weeks 2–4. A post-treatment 1 evaluation was conducted in week 5, followed by a rest period during weeks 6–7. During week 8, pre-treatment 2 evaluation was conducted, followed by the second treatment phase (weeks 9–11), and a post-treatment 2 evaluation (see Fig. 1).

### Baseline and outcome assessments

#### Baseline assessments

Participants underwent extensive language and neuropsychological testing at baseline. Testing included administration of the WAB-R, as well as a number of other cognitive-linguistic measures (Supplementary Table 1).^82^ In addition, biographical data were collected, including: date of stroke, stroke age, sex, history of or current diagnosis of depression, use of antidepressants, handedness, years of education, and number of days per week exercised at least 20 min (pre- and post-stroke). A comprehensive overview of neuropsychological test scores by treatment group is presented in Supplementary Table 2.

#### Outcome assessments

The Philadelphia Naming Test (PNT)^84^ served as the primary outcome measure for evaluating baseline anomia severity and treatment progress in un-trained naming. The PNT is a computer-based assessment of naming and includes 175 pictures representing low- to high-frequency nouns. To account for random variability in day-to-day performance, the PNT was administered twice at baseline and performance averaged across administrations. For assessment of progress, the PNT was administered immediately after the first treatment phase (week 5), before initiation of the second treatment phase (week 8; baseline assessment for second treatment), and immediately following the second treatment phase (week 12). Graduate research assistants, under the supervision of American Speech-Language-Hearing Association (ASHA)-certified Speech Language Pathologists (SLPs), scored and transcribed PNT assessments following testing guidelines. Scoring was conducted blinded to participant information and treatment order. Reliability and fidelity information on scoring procedures has been thoroughly described elsewhere.^82^^,^^85^

#### Treatment response

This study sample includes participants with varying degrees of naming deficits. For this reason, treatment-related improvement in naming was assessed as proportion of potential maximal gain score (PMG; for similar approaches, see refs. 22, 24 and 86). PMG outcomes correlate strongly with raw naming outcomes, but additionally offer an inherent control for baseline performance not captured by raw change in naming performance. This baseline adjustment enhances our sensitivity to detect meaningful effects of interest. For each participant, the change in PNT score from pre- to post-treatment was divided by the maximal potential change. The formula for calculating PMG is as follows:

[(Post-treatment PNT Score − pre-treatment PNT Score)/(175 − pre-treatment PNT Score)].

PMG was calculated separately for each treatment phase, for a total of two improvement scores per participant: Phonological treatment PMG (Phon PMG) and semantic treatment PMG (Sem PMG). Given a recent theoretical debate surrounding the validity of PMG as an outcome measure in stroke rehabilitation,^87^^,^^88^ all analyses reported herein were run on raw change scores as well. These results are reported in Supplementary materials.

### Treatment

This study employed an unblinded, cross-over design where all participants received 3 weeks of phonologically focussed treatment and 3 weeks of semantically focussed treatment. Treatment order was randomized for each participant using the asymptotic maximal procedure in a 1:1 ratio.^89^ Treatment was administered 5 days per week for ∼ 1 h per session, for a total of 15 treatment sessions for each treatment type. ASHA-certified SLPs with extensive experience in working with PWA administered therapy. Training of clinicians and monitoring of treatment delivery was conducted under the supervision of another ASHA-certified SLP. Monitoring of treatment fidelity involved observation of ∼10% of treatment sessions, as previously reported.^85^

#### Phonological treatment

Phonologically focussed treatment included three types of therapy tasks. The first is the phonological components analysis task.^66^ In this task, participants name a series of pictures and then identify phonological features of the target words (e.g. first sound, number of syllables, last sound, rhyming word, vowel in the first syllable and vowel in the last syllable). Second, participants completed a phonological production task.^90^^,^^91^ Participants are required to identify phonological features using a stack of imageable nouns and verbs. It requires the participant to first sort the stack of picture stimuli based on the number of syllables by tapping out each syllable. Once the participant has sorted the targeted words into two stacks, the treatment moves on to identifying the following hierarchy of phonological features using a pair of targeted nouns/verbs: (i) first syllable-first syllable; (ii) first syllable-last syllable; (iii) last syllable-last syllable; (iv) last syllable-first syllable; (v) first syllable-first sound; (vi) last syllable-last sound; (vii) first syllable-last sound; and (viii) last syllable-first sound. Once these features are identified for the pair of words, the participant is required to blend the syllables/sounds together. Last, participants completed a custom computerized phonological judgement task where they judged whether pairs of words (nouns and verbs) rhyme; are matched based on phonological features, including the same number of syllables, initial and final syllables; and compare which word has more syllables.

#### Semantic treatment

Semantically focussed treatment similarly employs three therapy tasks. The first task is semantic feature analysis (SFA).^52^^,^^92^ The participant sees a picture and is prompted to name the picture. Then, he or she is encouraged to produce semantically related words that represent features of the target word (e.g. superordinate category, use, action, physical properties, location and association). Regardless of naming accuracy on the last item, treatment continues on to the next stimulus item. Because both nouns and verbs have been used for SFA-focussed activities,^93^^,^^94^ stimuli for SFA tasks utilized both. Second, participants complete a semantic barrier task. This approach includes features of the Promoting Aphasics’ Communication Effectiveness (PACE) approach^95–98^ and has also been included as part of constraint-induced language therapy.^99^ It relies on a stack of picturable stimuli, which are split between the participant and clinician, and placed face up on a table. A visual barrier is placed between the clinician and the participant. The goal of the task is for the participant to describe each card using semantic features so that the other participant (e.g. clinician) can guess the picture on the card. The clinician models the kinds of cues that are allowed. The clinician and participant take turns describing pictures. The third approach, Verb Network Strengthening Treatment (VNeST), is a semantic treatment approach that targets lexical retrieval of verbs and their thematic nouns.^100^ The objective of VNeST is for the participant to generate verb–noun associates. VNeST can be modified to fit participants with very limited speech output (e.g. using sentence completion). Approximately 15 min were allocated to each therapy task within each treatment session, which allowed additional 15 min to orient the participant to treatment tasks, switching between tasks, and any unexpected delays.

To continue participant enrolment during the COVID-19 pandemic, the treatment protocol was modified for remote administration. Participants were mailed a teletherapy kit including a touchscreen laptop pre-loaded with the semantic and phonological treatment apps, Zoom for online videoconferencing with the SLP, and Team Viewer which allowed the SLP to see the participant’s screen to help with initial set-up and troubleshooting. The teletherapy kit also included a high-quality headset with a microphone to maximize quality of communication with the SLP, a mouse for optional use, and a mobile WiFi hotspot if a participant did not have adequate connection at home. Online treatment administration mimicked in-person administration as closely as reasonably possible. Observed treatment responses in the eleven participants who completed treatment remotely did not deviate from responses recorded in the participants who received in-person treatment.

### Neuroimaging

MRI data were acquired on a Siemens 3T MRI Prisma Fit scanner (Siemens Medical Systems, Erlangen, Germany) housed at the McCausland Center for Brain Imaging. A 20-channel head coil was used to acquire T_1_- (MP-RAGE: 1 mm isotropic voxels, matrix = 256 × 256, 9° flip angle) and T_2_-weighted images. Lesions were manually demarcated on T_2_ images by a licensed neurologist (author LB). MRI scans were not acquired for the participants who entered the study after the outbreak of COVID-19 due to local social distancing measures in place.

### Data analysis

#### Missing data

Less than 1.5% of baseline neuropsychological test scores were missing at random for the first 93 participants. Several neuropsychological tests were not administered to the remaining 11 participants who entered the study after the COVID-19 outbreak (see Supplementary Table 1). Less than 2.5% of baseline neuropsychological test scores were missing at random for the last 11 participants (excluding tests not administered). Therefore, the amount of available baseline data differed slightly depending on the parameters incorporated in reported statistical analyses; all such differences are detailed where applicable. Baseline PNT scores were missing for two participants and two participants were missing the second treatment follow-up scores due to video errors that affected offline scoring. These scores were imputed using the next available follow-up scores. Another three participants were missing PNTs from the first treatment follow-up. These scores were imputed by averaging the baseline and pre-treatment 2 scores.

#### Outliers

An initial visual inspection of participant data revealed five distinct outliers in the outcome variable (Supplementary Fig. 1). All five participants had PMG < −0.5 and Cook’s *D* > 0.1 (based on a reference regression model including five predictors), suggesting that including them in the analyses might substantially negate our power to detect meaningful predictors. Those participants were removed, leaving a total of 99 participants for data analysis.

### Statistical analysis

#### First aim

Consistent with the aims of the study, data analysis was performed in two stages. The first aim compares the effects of phonological and semantic treatment. Our primary statistical analysis included a linear mixed-effects model (LMM) examining the effects of treatment phase (first versus second) and treatment type (phonological versus semantic). To this end, the model included treatment phase (1/2), treatment type (Phon/Sem), and treatment order (Phon first/Sem first) as independent factors. Subject-specific random intercepts were incorporated. Phonological and semantic PMG outcomes served as the dependent variables. Aphasia severity was adjusted for by including WAB-AQ score as a covariate in the model. The LMM was run in R (version 3.6.0) using the *nmle* package (lme function). As a follow-up analysis, we ran *post hoc t*-tests to examine the within-group response to each treatment and to compare response to both treatments.

#### Second aim

The second aim was to identify predictors of within-group response to treatment, emphasizing factors that specifically predict response to phonological and semantic therapy. Given the discordant prior literature, we applied a data-driven approach to identify treatment predictors. To this end, we took several steps to identify predictive factors and reduce the risk of Type II Error. First, we ran a correlational analysis between Phon and Sem PMG outcomes and continuous baseline variables. A *P*-value of 0.01 or lower was considered indicative of statistical significance. Second, separate stepwise regression models incorporated continuous and categorical baseline variables as independent factors and Phon and Sem PMG outcomes as dependent factors (in separate models). Complementary to the correlational approach, this analysis served to identify the subset of predictors most strongly associated with treatment response when controlling for other predictors. Third, we used independent samples *t*-tests to investigate whether response to each treatment differed across categorical variables (e.g. fluent versus non-fluent speech, aphasia type, sex, etc.). Last, in an attempt to inform the question of whether treatment effects are maximized when treatment is administered in accordance with each participant’s level of breakdown or spared processing capacity, we examined (i) the association between measures of phonological {i.e. non-word repetition [Psycholinguistic Assessment of Language Processing in Aphasia (PALPA)^101^ 8]} and semantic processing [i.e. non-verbal semantic processing (PPTT^102^)], production of phonological and semantic errors on naming tasks, and good versus poor response to each treatment (PMG outcomes in quartiles 1 and 4), and (ii) the association between good versus poor performance on measures of phonological and semantic processing and good versus poor response to each treatment.

### Data availability

In accordance with the National Institute of Health policy for data sharing (http://grants.nih.gov/grants/policy/data_sharing/index.htm), upon completion of the POLAR trial and dissemination of primary study results, the analysis data files will be made available to the public, along with the final version of the study protocol, the data dictionary and brief instructions.

## Results

**Figure 2:**
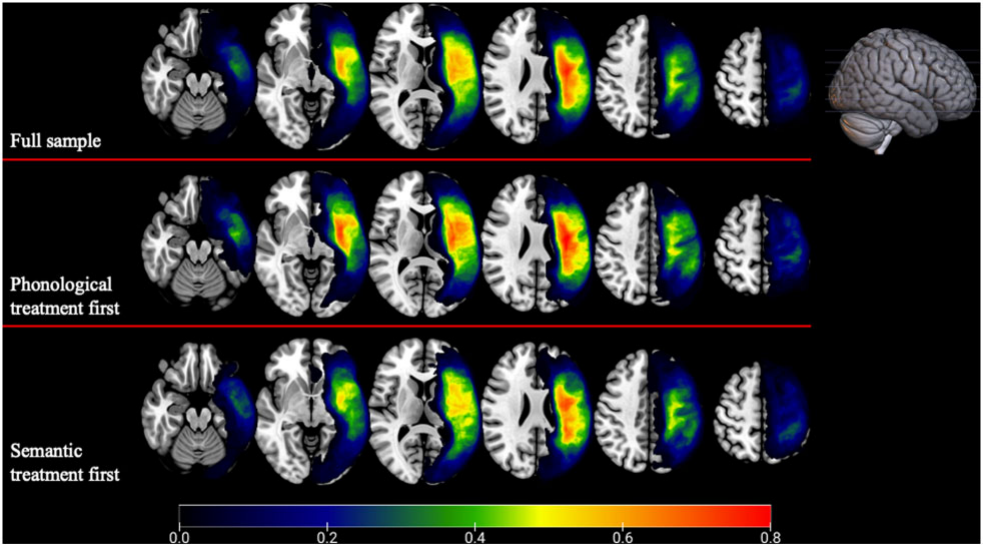
**Lesion overlap for study participants.** Overlap shown for full sample (max. overlap = 71/92), phonological treatment first group (max. overlap = 38/46), and semantic treatment first group (max. overlap = 33/46).

A total of 54 participants received the phonologically focussed treatment first (Phon first) and 50 participants received the semantically focussed treatment first (Sem first). As presented in Table 1, there were no differences in baseline characteristics across groups (*P* > 0.05 for all comparisons) for the 99 participants whose data were analysed. Lesion overlap centred around middle cerebral artery territory language regions in both groups, with maximal overlap in the Sem first group situated posterior to the maximal overlap in the Phon first group (Fig. 2).

### Aim 1: Between-group comparison of phonological versus semantic treatment effects

**Figure 3:**
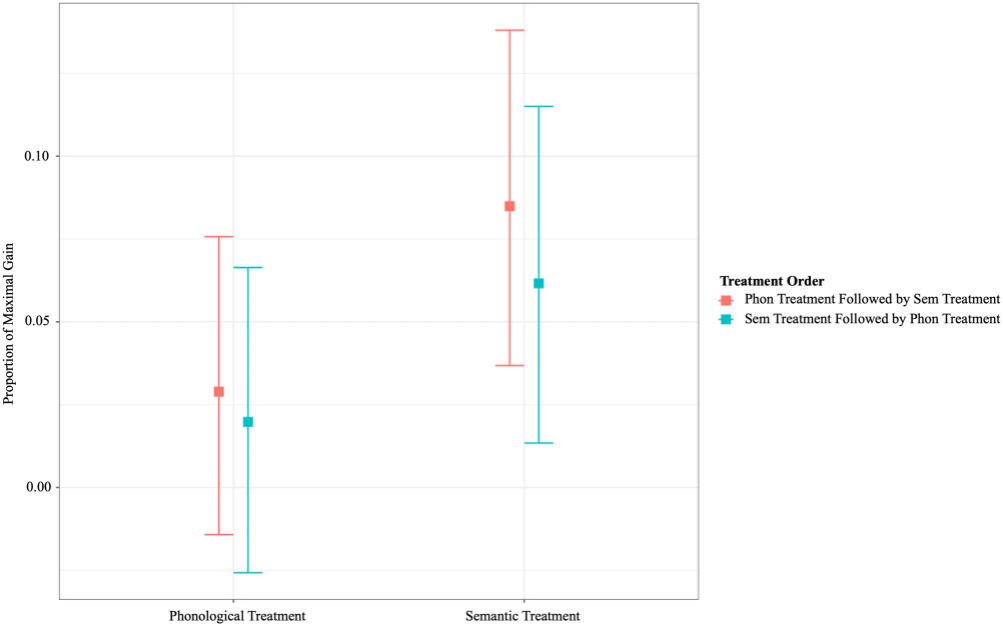
**Group-specific treatment effects.** Proportion of Maximal Gain by treatment type across treatment groups (phonological treatment first, *n* = 50; semantic treatment first, *n* = 49). Whiskers denote 95% confidence intervals of sample means.

Our primary analysis for comparing the effects of phonological and semantic treatment was an LMM. We found a significant effect of treatment type (reference level: semantic treatment; *β* = 0.048, *P* = 0.041), indicating that semantic treatment was significantly more beneficial than phonological treatment at the group-level across treatment groups (Fig. 3). Other factors did not reach statistical significance (treatment phase; *β* = 0.007, *P* = 0.768; treatment order, reference level: Sem first; *β* = −0.017, *P* = 0.467). Importantly, the treatment type term remained a significant predictor when the effects of aphasia severity (WAB-AQ; *β* = 0.002, *P* = 0.005) were accounted for.

**Figure 4:**
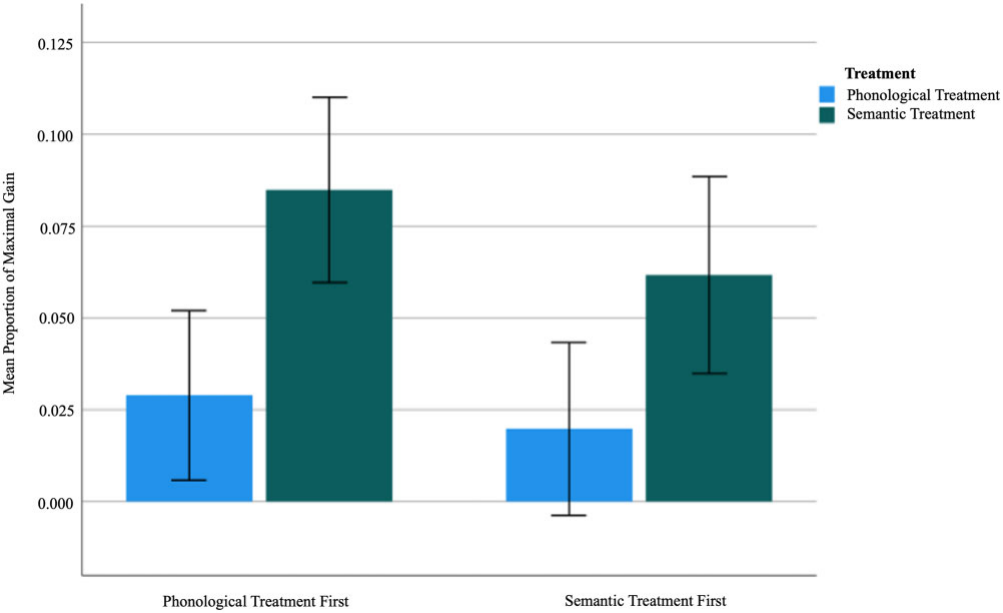
**Post-hoc comparison of recovery across groups.** Mean proportion of maximal gain following phonological and semantic treatment by treatment group (i.e. phonological treatment first, *n* = 50 versus semantic treatment first, *n* = 49). Whiskers show standard errors of means.

The within-group group analysis revealed that the Phon first group did not improve significantly in naming after the first treatment phase [PMG = 0.029, *t*(49) = 1.257, *P* = 0.108; Cohen’s *d* (*d*) = 0.18], whereas the Sem first group showed significant improvement [PMG = 0.062, *t*(48) = 2.300, *P* = 0.013; *d* = 0.33]. After the second treatment phase, the Phon first group showed a significant improvement in naming [PMG = 0.085, *t*(49) = 3.363, *P* = 0.001; *d* = 0.48], whereas the Sem first group did not [PMG = 0.020, *t*(48) = 0.844, *P* = 0.202; *d* = 0.12]. In other words, for both groups, the semantic treatment phase resulted in significantly improved PMG, whereas the phonological treatment phase did not. However, a paired-samples *t*-test revealed that there was not a statistically significant difference in the response to the first and second treatment phase for the Phon first group [PMG = 0.029 versus 0.085, *t*(49) = 1.766, *P* = 0.084; *d* = 0.25] or the Sem first group [PMG = 0.062 versus 0.020, *t*(48) = 1.086, *P* = 0.283; *d* = 0.16]. Effects of each treatment type by group are visualized in Fig. 4 and statistical tests are summarized in Table 2.

**Table 2:** Change by treatment order across treatment groups (phonological treatment first, *n* = 50; semantic treatment first, *n* = 49).

Treatment group	Assessment timepoint		Two-tailed *P*-value
	Treatment 1 PMG (one tailed *P*-value)	Treatment 2 PMG (one tailed *P*-value)	
Phonological treatment first	0.029 (0.108)	0.085 (0.001)	0.084
Semantic treatment first	0.062 (0.013)	0.020 (0.202)	0.283

Proportion of Maximal Gain was calculated based on change from baseline after each treatment period. One-tailed *P*-values in parentheses tested the hypothesis that there was a positive effect of treatment at a given timepoint [test statistics: phonological treatment first, *t*(49) = 1.257 and *t*(49) = 3.363 for treatment 1 and 2, respectively; semantic treatment first, *t*(48) = 2.300 and *t*(48) = 0.844 for treatment 1 and 2, respectively]. Two-tailed *P*-values tested whether treatment response differed across assessment timepoints within each group [phonological treatment first, paired-*t*(49) = 1.766; semantic treatment first, paired-*t*(48) = 1.086].

### Aim 2: Within-group predictors of treatment response

**Figure 5:**
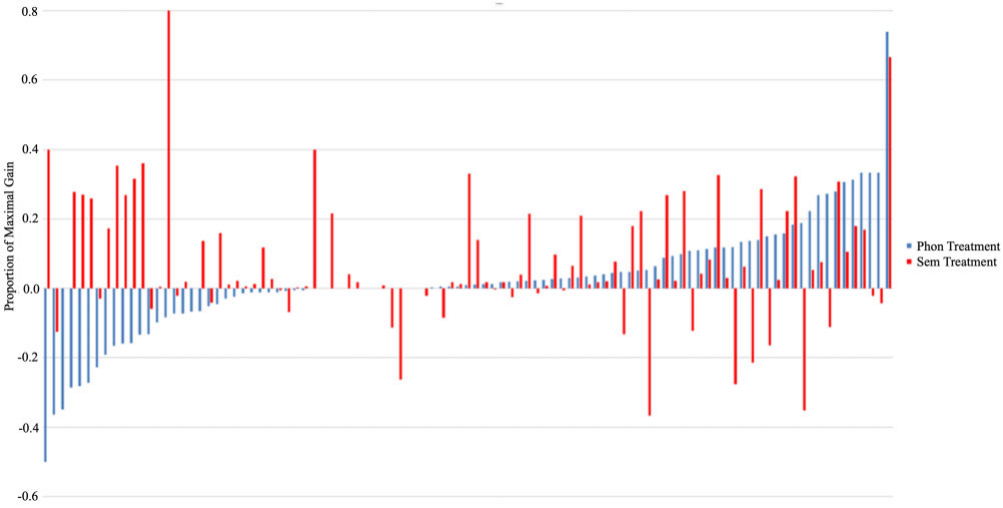
**Individual responses to phonological and semantic therapy.** Within-individual proportion of maximal gain (PMG) following phonological and semantic treatment, ordered from the lowest to highest phonological PMG and overlaid with semantic PMG (phonological treatment first, *n* = 50; semantic treatment first, *n* = 49).

Although the between-group analysis revealed a superior effect of the semantic treatment on average, some participants showed a clear benefit of the phonological treatment. Figure 5 shows the response to both treatments at the individual level, ordered from the least to greatest response to the phonological treatment and overlaid with the corresponding response to semantic treatment. As is clearly evident from Fig. 5, some participants respond to the phonological treatment and not the semantic treatment, some respond to semantic but not to phonological treatment, while others respond to neither or both treatments. The correlation between response to the phonological and semantic treatment was not significant (*r* = −0.016, *P* = 0.874).

Our first step in identifying factors associated with response to each treatment type was a correlational analysis. We ran a total of 58 pairwise correlations between Phon and Sem PMG outcomes and baseline neuropsychological variables. Given the data-driven design of the study, an uncorrected *P*-value of 0.01 was considered indicative of statistical significance. All significant pairwise correlations are shown in Table 3. Phon PMG was found to correlate significantly only with baseline NIHSS^103^ score (*r* = −0.301). Significant correlations were identified between Sem PMG and several subtests of the Western Aphasia Battery (*r* = 0.256–0.308), Naming 40 correct, an in-house assessment of mid- to high-frequency object naming (*r* = 0.339), several subtests of the Northwestern Assessment of Verbs in Sentences (NAVS^101^; *r* = 0.268–0.298), Temple Assessment of Language and Short-term Memory in Aphasia rhyming triplet score, a task that assess the ability to identify rhyming words out of a selection of three words (TALSA^104^; *r* = 0.291), subtest 8 (non-word repetition) of the PALPA^105^ (*r* = 0.373), and number of correctly named items on the baseline PNT (*r* = 0.276).

**Table 3:** Significant pairwise correlations between treatment response and baseline testing variables.

Variable	Semantic PMG	Phonological PMG
PALPA 8	0.373**	0.134
Naming 40 correct	0.339^*^	0.179
WAB Spontaneous Speech	0.308^*^	0.134
NIHSS	−0.077	−0.301^*^
NAVS Argument Structure Production Test	0.298^*^	0.056
TALSA Rhyming triplets	0.291^*^	0.039
WAB AQ	0.284^*^	0.092
PNT correct	0.276^*^	0.084
NAVS Argument Structure	0.273^*^	0.034
NAVS Sentence Comprehension Test	0.268^*^	0.059
WAB Naming	0.256^*^	0.083

NAVS, Northwestern Assessment of Verbs in Sentences; NIHSS, National Institute of Health Stroke Scale; PALPA, Psycholinguistic Assessment of Language Processing in Aphasia; PNT, Philadelphia Naming Test; TALSA, Temple Assessment of Language and Short-term Memory in Aphasia; WAB, Western Aphasia Battery (AQ: Aphasia Quotient).

* Significant Pearson’s correlation coefficients (*r*) at *P* < 0.01; **P < 0.001.

In the stepwise regression model for Sem PMG (adj. *R*^2^ = 0.353), WAB Spontaneous Speech (WAB-SS) score emerged as the best predictor [*β* = 0.52; *F*(1,63) = 14.6, *P* < 0.001, *R*^2^ = 0.19]. Other factors included in the final model were WAIS Matrix Reasoning^106^ [*β* = −0.33; *F*(2,62) = 10.9, *P* = 0.008, *R*^2^ = 0.07] score, a measure of non-verbal reasoning skills; self-reported number of days exercised per week prior to stroke-onset [*β* = −0.24; *F*(3,61) = 9.1, *P* = 0.023, *R*^2^ = 0.05]; number of mixed errors produced on the Philadelphia Repetition Test^84^ [*β* = −0.26; *F*(4,60) = 8.5, *P* = 0.016, *R*^2^ = 0.05]; and, number of semantically related errors produced on the PNT [*β* = −0.21; *F*(5,59) = 8.0, *P* = 0.045, *R*^2^ = 0.04] (see Table 4). The stepwise regression model for Phon PMG (adj. *R*^2^ = 0.218) retained three variables: NIHSS [*β* = −0.41; *F*(1,63) = 7.2, *P* = 0.001, *R*^2^ = 0.10]; lesion volume [*β* = 0.316; *F*(2,62) = 6.8, *P* = 0.010, *R*^2^ = 0.08]; and, self-reported antidepressants use [yes/no = 1/0; *β* = −0.27; *F*(3,61) = 6.9, *P* = 0.017, *R*^2^ = 0.07] (see Table 5).

**Table 4:** Stepwise regression model for post-semantic treatment PMG

Variable	Estimate	SE	*β*	*t*	*R^2^* change	Adj. *R^2^*	*P*-value
WAB-SS	0.021	0.005	0.521	4.327	0.188	0.175	<0.001
WAIS Matrix Reasoning	−0.011	0.004	−0.325	−2.736	0.072	0.236	0.008
Days of exercise prior to stroke	−0.019	0.008	−0.242	−2.341	0.049	0.275	0.023
PRT Mixed errors	−0.091	0.037	−0.257	−2.474	0.052	0.319	0.016
PNT Semantically related errors	−0.009	0.005	−0.209	−2.044	0.042	0.353	0.045

Stepping method criteria used probability of *F*: entry = 0.05, removal = 0.10. PNT, Philadelphia Naming Test; PRT, Philadelphia Repetition Test; SE, standard error; WAB-SS, Western Aphasia Battery Spontaneous Speech subtest; WAIS, Wechsler Adult Intelligence Scale.

**Table 5:** Stepwise regression model for post-phonological PMG

Variable	Estimate	SE	*β*	*t*	*R*^2^ change	Adj. *R*^2^	*P*-value
NIHSS	−0.017	0.005	−0.409	−3.431	0.103	0.089	0.001
Lesion volume	5.9*10^–7^	0.000	0.316	2.649	0.078	0.154	0.010
Antidepressants (Y/N; 1/0)	−0.095	0.039	−0.274	−2.458	0.074	0.218	0.017

Stepping method criteria used probability of *F*: entry = 0.05, removal = 0.10. Binary variables: reference level = 1. NIHSS, National Institute of Health Stroke Scale; SE, standard error.

Independent samples *t*-tests revealed that treatment response differed on a single variable, antidepressant use. Specifically, participants who reported that they regularly took antidepressants (type not specified) responded significantly worse to phonological treatment than their counterparts who did not use antidepressants [PMG: −0.03 versus 0.04, respectively, *t*(94) = 2.210, *P* = 0.03]. No such difference was observed for semantic treatment [PMG: users/non-users; 0.10 versus 0.06, *t*(94) = 0.943, *P* = 0.348]. In terms of aphasia type, greatest numerical difference was observed for anomic aphasia (Sem versus Phon PMG: 0.28 versus 0.13), but this difference failed to reach statistical significance [paired-*t*(24) = 1.238, *P* = 0.228]. The only statistically significant difference was observed in participants with conduction aphasia [Sem versus Phon PMG: 0.10 versus 0.01, paired-*t*(15) = 2.366, *P* = 0.032].

### Baseline characteristics associated with treatment-specific effects

A total of 54 participants showed a positive response to phonological treatment (IQR = 0.106), 64 showed a positive response to semantic treatment (IQR = 0.176), 38 participants responded to both treatments, and 18 to neither treatment (alternatively, 81 participants responded to at least one treatment). We took several additional steps to inform the question of who responds to what treatment. Consistent with our data-driven approach, the first analysis aimed to identify baseline predictors uniquely associated with response to each treatment type. To this end, we constructed stepwise linear regression models to predict phonological residuals (Phon Resid) after the effects of aphasia severity (WAB-AQ) and semantic treatment-specific effects (Sem PMG) had been accounted for. The variable Phon Resid was derived by predicting Phon PMG from WAB-AQ and Sem PMG, and subtracting actual form predicted Phon PMG. The same procedure was applied to derive semantic residuals (Sem Resid). Both residual scores were subsequently predicted from baseline measures that quantify semantic and phonological processing capacity (i.e. PPTT, PALPA, and semantic and phonological errors produced on the Naming 40, PRT, and PNT; see Supplementary Table 1). Sem Resid values were best predicted by a model containing a single variable: number of phonemic errors produced on the Naming 40 [*β* = −0.27; *F*(1,75) = 5.671, *P* = 0.020, *R*^2^ = 0.07]. No variables were retained in the model predicting Phon Resid values. The same approach was followed to predict treatment-specific residuals in participants who responded only to phonological (*n* = 16) and semantic (*n* = 27) treatment. In this case, the best model predicting Phon Resid (adj. *R*^2^ = 0.605) included number of phonemic errors produced on the Naming 40 [*β* = −0.87; *F*(1,12) = 10.158, *P* = 0.001, *R*^2^ = 0.46] and PALPA 15 [auditory rhyme judgement; *β* = 0.49; *F*(2,11) = 10.959, *P* = 0.024, *R*^2^ = 0.21]. The best model predicting Sem Resid (adj. *R*^2^ = 0.40) included number of semantic errors produced on the Naming 40 [*β* = −0.64; *F*(1,20) = 5.259, *P* = 0.002, *R*^2^ = 0.21] and PALPA 17 [segmentation of final sounds; *β* = 0.53; *F*(2,19) = 7.918, *P* = 0.009, *R*^2^ = 0.25].

Second, we examined performance on two measures representing phonological and semantic processing (PALPA 8—non-word repetition/PPTT—non-verbal semantic processing) and phonological and semantic errors produced on baseline naming tests in relation to whether participants showed a poor (first quartile; Q1) or good (Q4) response to each treatment. Compared to poor responders, participants who showed a good response to phonological treatment produced significantly more semantic errors on the Naming 40 [2.7 versus 1.8; *X*^2^(1, *N* = 39) = 4.509, *P* = 0.034] and fewer semantically unrelated errors on the PNT [3.7 versus 7.5; *X*^2^(1, *N* = 48) = 5.298, *P* = 0.021]. Similarly, participants who showed a good response to semantic treatment performed significantly better on PALPA 8 {non-word repetition; 13.9 versus 7.5; [*X*^2^(1, *N* = 43) = 7.667, *P* = 0.006]} and PPTT [47.3 versus 46.0; *X*^2^(1, *N* = 50) = 4.160, *P* = 0.041] than their counterparts who showed a poor response to semantic treatment.

Third, we reversed the question and analysed treatment response by performance on the PALPA 8 and PPTT (based on Q1 and Q4). Participants who performed best on the PPTT were more likely to respond to semantic treatment than participants who performed poorly on the PPTT [PMG: 0.13 versus 0.02; *X*^2^(1, *N* = 50) = 3.945, *P* = 0.047]. Participants who performed best on PALPA 8 similarly showed a substantially larger response to semantic treatment than participants whose performance was poorer [PMG: 0.19 versus 0.00; *X*^2^(1, *N* = 44) = 15.395, *P* < 0.001]. No differences were noted with regard to phonological treatment response.

Finally, given our findings that factors such as aphasia severity (WAB-AQ) and speech output (WAB-SS) predicted response to semantic treatment, whereas severity of stroke symptoms (NIHSS) and lesion volume predicted response to phonological treatment, we performed a *post hoc* analysis to examine the association between response to each treatment and presence or absence of apraxia of speech (AOS), and fluent or non-fluent speech production. Our results revealed that participants who showed a good response to phonological treatment were significantly more likely to present with AOS compared to poor responders [*n*: 15 versus 7; *X*^2^(1, *N* = 48) = 5.371, *P* = 0.020]. On the contrary, participants who showed a good response to semantic treatment were significantly more likely to present with fluent speech compared to poor responders [*n*: 19 versus 10; *X*^2^(1, *N* = 49) = 7.776, *P* = 0.005]. These results are summarized in Table 6.

**Table 6:** Predictors of treatment-specific proportion of maximum gain colligated across statistical analyses

	Treatment		
Measure	Phonological treatment response	Semantic treatment response	Implications
Phonological processing	(i) SWR (DV: Phon Resid, *n* = 16) identified PALPA 15 score (*β* = 0.49) when # of phonemic errors on N40 was accounted for.	SWR (DV: Sem Resid, *n* = 27) identified PALPA 17 score (*β* = 0.53) when # of semantic errors on N40 was accounted for. Responders (Q4) scored higher on PALPA 8 than non-responders (Q1) (*n* = 43, *P* < 0.01). (iii) High scorers on PALPA 8 (Q4) were more likely to be responders than low scorers (Q1) (*n* = 44, *P* < 0.001).	Preserved phonological processing skills may predict favourable response to semantic treatment; the association between phonological processing skills and response to phonological treatment is less clear.
# of phonological speech errors	(i) SWR (DV: Phon Resid, *n* = 16) identified # of errors on N40 (*β* = −0.87) when PALPA 15 score was accounted for.	(i) SWR (DV: Sem Resid, *n* = 99) identified # of errors on N40 (*β* = −0.27).	More phonological speech errors produced on naming tasks may negate response to both treatments to some degree.
Semantic processing		Responders (Q4) scored higher on PPTT than non-responders (Q1) (*n* = 50, *P* < 0.05). High scorers on PPTT (Q4) were more likely to be responders than low scorers (Q1) (*n* = 50, *P* < 0.05).	Preserved semantic processing skills may be associated with favourable response to semantic treatment.
# of semantic speech errors	Responders (Q4) produced more errors on N40 than non-responders (Q1) (*n* = 39, *P* < 0.05). Responders (Q4) produced fewer unrelated errors on PNT than non-responders (Q1) (*n* = 48, *P* < 0.05).	(i) SWR (DV: Sem Resid, *n* = 27) identified # of errors on N40 (*β* = −0.64) when PALPA 17 score was accounted for.	The number of semantic speech errors may be positively associated with response to phonological treatment and negatively with response to semantic treatment.
Apraxia of Speech	(i) Responders (Q4) were more likely to present with apraxia of speech than non-responders (Q1) (*n* = 48, *P* < 0.05).		Presence of apraxia of speech may be associated with response to phonological treatment.
Fluency		(i) Responders (Q4) were more likely to present with fluent speech than non-responders (Q1) (*n* = 49, *P* < 0.01).	Fluent speech production may be associated with response to semantic treatment.

DV, dependent variable; N40, Naming 40; PALPA, Psycholinguistic Assessment of Language Processing in Aphasia (PALPA 15: auditory rhyme judgement; PALPA 17: segmentation of final sounds; PALPA 8: non-word repetition); PNT, Philadelphia Naming Test; PPTT, Pyramids and Palm Trees Test; Q, quartile (1: first quartile; 4: fourth quartile); Sem/Phon Resid, residuals of semantic/phonological proportion of maximum gain regressed on Western Aphasia Battery Aphasia Quotient and phonological/semantic proportion of maximum gain; SWR, stepwise regression.

## Discussion

This study compared the effects of phonologically and semantically focussed aphasia treatment and examined biographical and neuropsychological predictors of treatment response in a sample of 99 participants recruited for the POLAR trial. Our primary results revealed a significant benefit of semantically focussed treatment at the group-level. Notwithstanding, treatment response varied considerably at the individual level. Spontaneous speech performance emerged as the strongest independent predictor of semantic treatment response and the degree of recovery was positively associated with non-word repetition, naming, syntactic processing, and overall severity of aphasia. Severity of stroke symptoms emerged as the strongest predictor of response to phonological treatment, and lesion volume and use of antidepressants improved prediction accuracy. Semantic treatment responders were characterized by the good performance on semantic and phonological baseline tasks, and fluent speech production. A less clear pattern was observed in phonological treatment responders; nonetheless, responders were found to be more likely to present with AOS compared to non-responders. These findings are discussed below, and future directions postulated.

Our study was motivated by the fundamental question of how to personalize aphasia treatment (i.e. semantic versus phonological treatment) based on baseline performance on commonly available and widely used cognitive-linguistic measures. As a first step, we aimed to compare the effects of each treatment to examine if one treatment yielded greater naming improvements in a minimally restricted sample of PWA. Contrary to several prior studies,^38^^,^^65^^,^^107^^,^^108^ we found a significant benefit of the semantically focussed treatment. Our linear mixed-effect model revealed that this effect was constant independent of which treatment was administered first. In fact, our within-group analysis indicated that both groups improved significantly after semantic treatment (Phon first = 0.085, *P* = 0.001; Sem first = 0.062, *P* = 0.013), while neither group improved after the phonological treatment (Phon first = 0.029, *P* = 0.108; Sem first = 0.020, *P* = 0.202). The use of *proportion of maximal gain* as an outcome measure has been criticized for inducing bias by inflating change scores for participants with mild aphasia.^87^^,^^109^ Therefore, we ran all reported analyses on raw change scores as well. Importantly, treatment type was similarly a significant predictor of raw change (reference level: semantic treatment; *β* = 2.34, *P* = 0.036; Supplementary Table 3), i.e. in favour of semantic treatment. The within-group analysis yielded comparable results with respect to semantic treatment (Phon first = 4.60, *P* = 0.001; Sem first = 3.93, *P* < 0.001). However, the raw data analysis additionally revealed a significant improvement after phonological treatment in the Phon first group only (2.15, *P* = 0.008; Supplementary Table 4). Thus, the raw analyses confirm the conclusion that semantic treatment is more beneficial on average at the group-level, but nonetheless suggest somewhat stronger effects of phonological treatment within one of two treatment groups.

In the interest of advancing knowledge on personalized treatment in aphasia, we furthermore aimed to identify predictors of treatment response. Prior work investigating whether anomia treatment should focus on the locus of breakdown (phonological versus semantic deficit) or relatively spared processes has consistently failed to find clear relationship between deficit profiles and response to specific treatment.^38–40^^,^^42^ Given the inconsistent prior findings, we opted for a data-driven approach where all baseline measures were given equal weight, followed by specific consideration of measures of phonological and semantic processing. To this end, our results revealed several interesting findings. First, it was not the case that some participants improved while others did not, regardless of which treatment was administered. The correlation between response to phonological and semantic treatment was insignificant (*P* = 0.874), and Fig. 5 shows variable response profiles across participants. Critically, this finding indicates that the efficacy of anomia treatment cannot be assumed to be comparable for any given individual; on the contrary, individuals seem to respond differently to phonological and semantic treatment.

Second, the degree of improvement following semantic treatment was associated with performance on multiple neuropsychological tests, including naming, speech output, syntactic processing and auditory short-term memory. All correlations were positive, indicating that a milder language deficit is associated with greater treatment progress. These findings are consistent with prior findings suggesting that individuals with milder aphasia generally respond better to treatment.^24^^,^^25^^,^^110^ Similar relationships were not noted for the degree of improvement following phonological treatment. Instead, the only significant relationship observed was a negative correlation with severity of stroke symptoms, suggesting that a greater overall disability may be associated with poor response to phonological treatment.

Third, the Spontaneous Speech subtest of the WAB-R emerged as the strongest positive predictor of semantic treatment response, accounting for 19% of the variability. WAIS Matrix Reasoning score, a measure of non-verbal reasoning skills, and number of days exercised prior to stroke were negative predictors in the same model. It is unclear if and how these factors may contribute to recovery. Of note, amount of exercise was self-reported and, as such, may be prone to subjective definitions across participants. In terms of reasoning skills, prior studies have not revealed a similar relationship. On the contrary, prior studies have suggested a positive relationship between cognitive functioning and treatment response.^22^ Therefore, these predictors should be interpreted with caution. Mixed and semantically related errors produced on the PRT and PNT, respectively, also emerged as negative predictors. Adjusting for the contribution of other predictors in the model, more errors produced seem to negate the degree of recovery. Interestingly, our results also revealed that participants with conduction aphasia showed a much greater response to semantic compared to phonological treatment. Anomic participants similarly showed a greater response to semantic treatment, although statistical significance was not observed. While recent evidence has suggested that aphasia research should place minimal focus on typological classification of aphasia syndromes,^111–117^ our results suggest that aphasia typology (i.e. aphasia type or fluent/non-fluent aphasia) may be a useful tool for guiding treatment planning in clinical practice.

Fourth, fewer robust baseline predictors of phonological treatment response were identified. Specifically, severity of stroke symptoms had the strongest predictive value, followed by lesion volume, and use of antidepressants. The inverse relationship between stroke symptoms and treatment response is not surprising; NIHSS score correlated significantly with both lesion volume (*r* = 0.310, *P* < 0.01) and WAB-AQ (*r* = −0.598, *P* < 0.01), and has been associated with poor treatment response in prior studies.^7^ Lesion volume was positively associated with outcome, suggesting greater recovery in participants with larger lesions. In general, this seems unlikely from a biological perspective. A more probable explanation relates to the fact that variability explained by stroke symptoms, which correlated with lesion volume, had already been accounted for; thus, the effects of lesion volume may instead indicate a somewhat contradictory association where larger lesions support favourable recovery in some individuals. This notion is supported by the finding that participants who show a good response to phonological treatment are more likely to present with AOS than those who respond poorly to treatment, as participants with AOS had larger lesions than participants without AOS [143 versus 96 cc; *t*(90) = 2.566, *P* = 0.012]. To this end, the actual relationship may be reversed, i.e. individuals with AOS adjuvant to aphasia may be more likely to respond to phonological treatment than those without AOS. Last, use of antidepressants negatively impacted treatment response. A *post hoc t*-test revealed that participants who reported using antidepressants responded worse to phonological treatment than those who did not report use of antidepressants [PMG: −0.03 versus 0.04; *t*(94) = 2.210, *P* = 0.030]. A considerable body of literature has investigated this relationship,^118^ but in this case, it is important to note that we did not collect data on drug type or dosage. Therefore, the effect of antidepressant use requires scrutiny in future research.

Finally, we examined the relationship between treatment response and phonological and semantic processing deficit profiles. Our intention was to inform the debate on whether treatment should be applied in line with individuals’ level of breakdown or spared processing. Given the mixed findings reported in smaller samples, we relied on hypothesis-free statistical procedures. Briefly, we found an association between the number of both phonological and semantic speech errors produced and response to phonological treatment. The nature of the relationship was elusive; positive response was associated with fewer or more errors across tasks. Error production was found to be highly confounded with presence/absence of AOS, in particular production of phonological speech errors. As such, assigning production errors to a breakdown at the level of phonological or semantic processing would be an inherently inaccurate undertaking. As an interim conclusion, we did not find a strong relationship between deficit profiles and response to phonological treatment. Future research efforts will be required to dissociate and disseminate the independent effects of the presence or severity of AOS and speech production errors on treatment response.

In terms of semantic treatment, we found that good response to treatment was associated with preserved phonological and semantic processing and fewer speech errors. While these findings hardly offer concrete conclusions regarding whether treatment should focus on the level of breakdown or spared processing, they do manifest in the notion alluded to above that individuals with less severe aphasia respond favourably to semantic treatment. Further supporting this conclusion, responders were more likely to present with fluent speech production, but fluent speech was highly confounded with aphasia severity [fluent versus non-fluent WAB-AQ; 70.3 versus 47.0, *t*(96) = 5.993, *P* < 0.001]. This finding needs to be replicated and future studies should aim to characterize in greater detail the relationship between severity and response to semantic treatment. The latter question is an intriguing one with reference to the relationship between AOS, lesion volume, and stroke symptoms for phonological treatment response. A recent study by Pompon et al.^119^ observed an inverse relationship between aphasia severity and the effects of phonomotor treatment in a sample of 26 participants, suggesting that severity may critically affect response to phonological treatment as well. To this end, future hypothesis testing comparing response to phonological and semantic treatment will certainly need to account for indices of symptom severity.

There are several important limitations to be considered when interpreting our findings. In terms of comparing our findings to the relevant literature, the most obvious limitation is that we did not classify participants based on whether they had primary phonological or semantic deficit profiles.^38^^,^^39^^,^^107^ The reason is simply that it is notoriously difficult, and perhaps unreasonably presumptuous, to categorize participants with these labels.^40^^,^^45^^,^^107^ A prime example of this was the exclusion of 29/87 participants in Doesborgh et al.’s^107^ study due to the fact that these participants could not be classified as presenting with primary phonological and/or semantic deficits. Rather, we focussed on including a clinically representative sample of participants. Second, the semantic and phonological treatment paradigms are not without limitations. The tasks applied in our semantic treatment relied heavily on novel speech productions, whereas the latter two tasks in our phonological treatment relied more on higher order phonological processing. Thus, while both treatments relied on widely recognized approaches shown to induce gains in naming performance, our results should be interpreted with reference to the ‘active ingredients’ in each treatment. A third limitation is that we did not assess improvements in phonological and semantic processing. It is entirely possible that participants improved in these functional domains after the respective treatment. Furthermore, the data-driven approach implemented here has some important limitations, including the large number of statistical tests conducted. Nonetheless, we would argue that the disparate prior literature highlights the need for a hypothesis-free examination. Given that a comparable study has not been conducted in a sample this large before, our findings are ideally suited for subsequent hypothesis testing in future studies. Last, we did not assess maintenance of treatment gains in the long-term. One of the primary motivations underlying phonological treatments is to re-establish connections between the building blocks of language (phonemes), with the intention of promoting generalization and lasting gains.^108^^,^^119^ Future studies should aim to decipher whether this proves to be the case.

## Conclusion

Our study compared and predicted response to phonological and semantic treatment in chronic aphasia. The fundamental question of who benefits from what type of treatment has puzzled clinicians and researchers in our field for a long time, and there has been a notable lack of large-scale rigorous treatment studies addressing this issue. Our findings do not support the notion that treatment allocation has a one-size-fits-all solution, nor that phonological deficits predict good response to phonological treatment and semantic deficits predict good response to semantic treatment. Instead, a comprehensive view of our results indicates that individuals with relatively mild aphasia benefit from semantic treatment, while those with more severe deficits may respond better to phonological treatment. The mechanistic account underlying treatment response and the minimally clinically important difference that distinguishes responders to each treatment remain topics for further study.

## Supplementary material

Supplementary material is available at *Brain Communications* online.

## Supplementary Material

fcab174_Supplementary_Data

## Abbreviations

AOS =: apraxia of speech
AQ =: aphasia quotient
ASHA =: American Speech-Language-Hearing Association
LMM =: linear mixed-effects model
MUSC =: Medical University of South Carolina
NAVS =: Northwestern Assessment of Verbs in Sentences
NIHSS =: National Institute of Health Stroke Scale
PACE =: Promoting Aphasics’ Communication Effectiveness
PALPA =: Psycholinguistic Assessment of Language Processing in Aphasia
PMG =: proportion of potential maximal gain
PNT =: Philadelphia Naming Test
POLAR =: Predicting Outcome of Language Rehabilitation in Aphasia
PPTT =: Pyramids and Palm Trees Test
PRT =: Philadelphia Repetition Test
PWA =: persons with aphasia
Q1 =: First Quartile
Q4 =: Fourth Quartile
SFA =: Semantic Feature Analysis
SLP =: Speech-Language Pathologist
SS =: Spontaneous Speech
TALSA =: Temple Assessment of Language and Short-term Memory in Aphasia
USC =: University of South Carolina
VNeST =: Verb Network Strengthening Treatment
WAB-R =: Western Aphasia Battery-Revised
WAIS =: Wechsler Adult Intelligence Scale
