# Supplementary material for: Individualized response to semantic versus phonological aphasia therapies in stroke
Source: Brain Commun. 2021 Aug 5;3(3):fcab174. doi: 10.1093/braincomms/fcab174 (PMC8376685; doi:10.1093/braincomms/fcab174)
Supplement: fcab174_Supplementary_Data [file fcab174_supplementary_data.pdf]

Supplementary Material

Tables

Assessment	Measure	Timepoint	Reference
ASRS	Presence and severity of apraxia of speech.	Baseline (week 1)	Strand, Duffy, Clark, & Josephs (2014).
Discourse tasks (Broken Window picture description, Cinderella story retelling, PBJ procedural description)	Discourse.	Baseline (week 1)<br>Post-Tx 1 (week 5)<br>Inter-Tx (week 8)<br>Post-Tx 2 (week 12)	Menn, Ramsberger, & Estabrooks (1994); Grimes (2005); Lau (2013).
KDT	Semantic processing of verbs.	Baseline (week 1)	Bak (2003).
*Naming 40	Naming.	Baseline (week 1)<br>Post-Tx 1 (week 5)<br>Inter-Tx (week 8)<br>Post-Tx 2 (week 12)	In-house.
NIHSS	Severity of stroke symptoms.	Baseline (week 1)	Brott et al. (1989).
NAVS	Syntactic processing.	Baseline (week 1)	Cho-Reyes & Thompson (2012).
PNT	Naming.	Baseline (week 1)<br>Post-Tx 1 (week 5)<br>Inter-Tx (week 8)<br>Post-Tx 2 (week 12)	Roach et al. (1996).
PRT	Repetition.	Baseline (week 1)	Roach et al. (1996).
*PALPA Subtest 1	Phonological processing; nonword discrimination.	Baseline (week 1)	Kay, Lesser, & Coltheart (2009).
*PALPA Subtest 2	Phonological processing; real word discrimination.	-	-
*PALPA Subtest 8	Phonological processing; nonword repetition.	-	-
*PALPA Subtest 14	Phonological processing; silent rhyme judgement.	-	-
*PALPA Subtest 15	Phonological processing; auditory rhyme judgement.	-	-

*PALPA Subtest 16	Phonological processing; segmentation of initial sounds.	-	-
*PALPA Subtest 17	Phonological processing; segmentation of final sounds.	-	-
PPTT	Semantic processing of nouns.	Baseline (week 1)	Howard & Patterson (1992).
*TALSA	Auditory short-term memory.	Baseline (week 1)	Martin et al. (2012).
WAIS	Nonverbal reasoning.	Baseline (week 1)	Wechsler (2008).
WAB-R	Aphasia severity.	Baseline (week 1).	Kertesz (2007).

Supplementary Table 1. Neuropsychological test batteries. Test abbreviations: ASRS = Apraxia of Speech Rating Scale; KDT = Kissing and Dancing Test; NIHSS = National Institute of Health Stroke Scale; NAVS = Northwestern Assessment of Verbs in Sentences; PNT = Philadelphia Naming Test; PRT = Philadelphia Repetition Test; PALPA = Psycholinguistic Assessment of Language Processing in Aphasia; PPTT = Pyramids and Palm Trees Test; TALSA = Temple Assessment of Language and Short-Term Memory in Aphasia; WAIS = Wechsler Adult Intelligence Scale; WAB-R = Western Aphasia Battery-Revised. * Not administered to N = 11 participants who entered the study after the outbreak of Covid-19.

Measure	Treatment group

	Phonological first (n = 50)	n	Semantic first (n = 49)	n	Two-tailed *p*-value[a]
WAB					
Spontaneous Speech	11.22 (SD = 4.62)	50	11.45 (SD = 4.81)	49	.810
Auditory Compreh.	7.90 (SD = 1.70)	50	7.46 (SD = 1.87)	49	.224
Repetition	4.81 (SD = 2.78)	50	5.48 (SD = 2.88)	49	.238
Naming	5.39 (SD = 3.06)	50	5.41 (SD = 2.84)	49	.973
ASRS Apraxia of Speech score	1.74 (SD = 1.61)	50	1.64 (SD = 1.52)	47	.751
WAIS	11.96 (SD = 6.31)	50	11.86 (SD = 5.12)	49	.929
PPTT	45.42 (SD = 4.86)	50	44.69 (SD = 6.75)	49	.540
KDT	46.08 (SD = 4.58)	50	45.39 (SD = 6.89)	49	.557
PRT	101.56 (SD = 58.35)	50	114.50 (SD = 52.83)	48	.253
Naming 40	15.88 (SD = 13.62)	40	15.59 (SD = 12.77)	44	.922
NAVS subscores					
VNT	10.13 (SD = 7.60)	48	9.94 (SD = 7.20)	47	.901
VCT	19.02 (SD = 4.01)	50	19.13 (SD = 3.94)	48	.896
Argument Structure	17.24 (SD = 12.09)	46	16.00 (SD = 13.31)	47	.640
ASPT	15.41 (SD = 11.96)	46	16.09 (SD = 13.31)	46	.799
SPPT	8.78 (SD = 9.78)	46	9.70 (SD = 11.05)	46	.676
SCT	22.08 (SD = 5.86)	50	20.77 (SD = 5.84)	48	.271
TALSA					
W5SF	12.98 (SD = 3.05)	43	13.33 (SD = 3.25)	46	.603
W5SU	14.67 (SD = 3.49)	43	15.07 (SD = 3.40)	46	.594
NW5SF	13.69 (SD = 2.77)	42	13.46 (SD = 3.02)	46	.707
NW5SU	14.02 (SD = 3.19)	42	14.61 (SD = 3.44)	46	.412

Triplet 1	9.65 (SD = 2.94)	43	9.43 (SD = 3.35)	46	.748
Triplet 2	9.37 (SD = 3.12)	43	8.98 (SD = 3.28)	46	.563
PALPA					
PALPA 1	57.33 (SD = 11.35)	43	58.50 (SD = 7.28)	46	.560
PALPA 2	59.21 (SD = 10.17)	43	60.13 (SD = 9.31)	46	.657
PALPA 8	6.42 (SD = 6.83)	43	8.26 (SD = 7.49)	46	.230
PALPA 14	9.19 (SD = 3.92)	43	10.15 (SD = 4.10)	46	.259
PALPA 15	46.49 (SD = 9.01)	43	48.50 (SD = 7.12)	46	.244
PALPA 16 ISW	18.05 (SD = 7.24)	43	17.93 (SD = 6.77)	43	.939
PALPA 16 ISNW	7.84 (SD = 3.66)	43	7.88 (SD = 3.42)	43	.952
PALPA 17 FSW	13.10 (SD = 6.54)	42	12.88 (SD = 7.47)	43	.890
PALPA 17 FSNW	6.40 (SD = 3.69)	42	6.60 (SD = 4.12)	43	.814
Discourse					
Mean CWPM	44.99 (SD = 31.68)	50	48.63 (SD = 37.13)	48	.602
Mean PD	.39 (SD = .16)	50	.39 (SD = .14)	48	.951
Mean VPU	.71 (SD = .50)	50	.82 (SD = .54)	48	.327

Supplementary Table 2. All neuropsychological test scores across treatment groups. ASRS = Apraxia of Speech Rating Scale; CWPM = content words per minute; KDT = Kissing and Dancing Test; NAVS = Northwestern Assessment of Verbs in Sentences (VNT: verb naming test; VCT: verb comprehension test; ASPT: argument structure production test; SPPT: sentence priming production test; SCT: sentence comprehension test); PALPA = Psycholinguistic Assessment of Language Processing in Aphasia (ISW and ISNW: segmentation of initial sounds in words and nonwords, respectively; FSW and FSNW: segmentation of final sounds in words and nonwords, respectively); PD = propositional density; PPTT = Pyramids and Palm Trees Test; PRT = Philadelphia Repetition Test; TALSA = Temple Assessment of Language and Short-term Memory in Aphasia (W5SF and W5SU: words 5-second filled and unfilled, respectively; NW5SF and NW5SU: nonwords 5-second filled and unfilled, respectively); VPU = verbs per utterance; WAB = Western Aphasia Battery; WAIS = Wechsler Adult Intelligence Scale.
[a]Independent samples *t*-tests were used for all comparisons, unless otherwise denoted.

Supplementary Results

The use of proportion of maximum gain as an outcome measure in stroke and aphasia rehabilitation literature has been criticized for inducing biased results by inflating observed pre- to post-treatment scores for participants with mild symptoms (as discussed in the manuscript). In an effort to fully disclose our findings, we herein report supplementary results based on raw PNT change scores. Specifically, we implemented the same statistical procedures as described in the manuscript on raw scores instead of PMG. These results should be considered supplementary and these findings should be interpreted in parallel with the PMG findings, and vice versa.

Tables

Fixed effects	Estimate	Standard Error	df	*t*-value	Pr (>|t|)
Intercept	0.33	2.61	97	0.13	0.897
Tx Type	2.34	1.13	97	2.08	0.036*
Tx Phase	0.11	1.13	97	0.10	0.923
Tx Order	-0.59	1.27	96	-0.46	0.640
WAB-AQ	0.03	0.03	96	1.02	0.302

Supplementary Table 3. Linear mixed-effects model for raw PNT change scores. Random subject-specific intercepts. Model AIC-value (Akaike Information Criterion) = 1415.0. Reference levels: Tx Tye = Semantic treaetment; Tx Order = Semantic treatment followed by phonological treatment. Tx = treatment; WAB-AQ = Western Aphasia Battery Aphasia Quotient. * $p < .05$.

Treatment group	Assessment timepoint		Two-tailed *p*-value
	Post Treatment 1 (one tailed *p*-value)	Post Treatment 2 (one tailed *p*-value)	
Phonological treatment first	2.15 (.008)<br>*d* = .35	4.60 (.001)<br>*d* = .45	.154<br>*d* = .21
Semantic treatment first	3.93 (<.001)<br>*d* = .48	1.69 (.095)<br>*d* = .19	.138<br>*d* = .22

***Supplementary* Table 4.** Change by treatment order across treatment groups (phonological treatment first, n=50; semantic treatment first, n=49). Raw change was calculated based on change from baseline after each treatment period. One-tailed p-values in parentheses tested the hypothesis that there was a positive effect of treatment at a given timepoint (test statistics: phonological treatment first, t(49)=2.486 and t(49)=3.193 for treatment 1 and 2, respectively; semantic treatment first, t(48)=3.382 and t(48)=1.332 for treatment 1 and 2, respectively). Two-tailed p-values tested whether treatment response differed across assessment timepoints (phonological treatment first, paired-t(49)=1.447; semantic treatment first, paired-t(48)=1.510).

Outcome	Post-Phon Tx raw change	Post-Sem Tx raw change
Post-Phon Tx raw change	--	.127 (p = .209)
Post-Sem Tx raw change	.127 (p = .209)	--

Supplementary Table 5. Pearson's correlation coefficient for raw PNT change scores following phonological and semantic treatment. Two-tailed p-value is reported in parentheses. Post-Phon Tx = Post-phonological treatment; Post-Sem Tx = Post-semantic treatment.

Variable	Post-Sem Tx raw change	Post-Phon Tx raw change
PALPA 8	.145	.042
Naming 40 correct	.114	.086
WAB Spontaneous Speech	.095	.113
NIHSS	.055	-.163
NAVS Argument Structure Production Test	.115	.013
TALSA Rhyming triplets	.093	-.001
WAB AQ	.070	.085
PNT correct	.045	.022
NAVS Argument Structure	.115	.009
NAVS Sentence Comprehension Test	.120	.064
WAB Naming	.042	.036

Supplementary Table 6. Correlations between phonological and semantic raw PNT change scores and variables identified as significantly correlated with phonological and semantic proportion of maximal gain (PMG). NAVS = Northwestern Assessment of Verbs in Sentences; NIHSS = National Institute of Health Stroke Scale; PALPA = Psycholinguistic Assessment of Language Processing in Aphasia; PNT = Philadelphia Naming Test; Post-Phon Tx = Post-phonological treatment; Post-Sem Tx = Post-semantic treatment; TALSA = Temple Assessment of Language and Short-term Memory in Aphasia; WAB = Western Aphasia Battery (AQ: Aphasia Quotient). An uncorrected $p < .01$ was considered indicative of statistical significance.

Variable	Post-Sem Tx raw change	Post-Phon Tx raw change
ASRS Apraxia of Speech	.017	.229*
PNT semantically related errors	-.028	.221*
PNT mixed errors	.046	.201*

Supplementary Table 7. Significant pairwise correlations between raw PNT change scores and baseline testing variables. ASRS = Apraxia of Speech Rating Scale; PNT = Philadelphia Naming Test; Post-Phon Tx = Post-phonological treatment; Post-Sem Tx = Post-semantic treatment. * denotes significant Pearson's correlation coefficients (r) at an uncorrected $p < .05$.

Variable	Estimate	SE	ß	*t*	R^2 change	Adj. R^2	*p*-value
ASRS Apraxia of Speech (Y/N; 1/0)	6.356	1.737	.399	3.659	.120	.106	.001
PPTT	.409	.134	.333	3.056	.112	.207	.003
PNT semantically related errors	.453	.192	.253	2.356	.064	.262	.022

Supplementary Table 8. Stepwise regression model for post-phonological treatment raw PNT change score. Stepping method criteria used probability of F: entry = .05, removal = .10. Binary variables: reference level = 1. ASRS = Apraxia of Speech Rating Scale; PNT = Philadelphia Naming Test; PPTT = Pyramids and Palm Trees Test; SE = standard error.

Variable	Estimate	SE	ß	*t*	R^2 change	Adj. R^2	*p*-value
	--	--	--	--	--	--	--

Supplementary Table 9. Stepwise regression model for post-semantic treatment raw PNT change score. Stepping method criteria used probability of F: entry = .05, removal = .10. No variables were included in the final model.

Treatment/ antidepressant use	n	Mean PNT change	SE	df	|t|	Two-tailed *p*-value
**Post-phonological treatment**						
No	66	2.3	1.00	94	1.273	.206
Yes	30	.2	1.08			
**Post-semantic treatment**						
No	66	4.9	1.18	94	1.107	.271
Yes	30	2.7	1.51			

Supplementary Table 10. Independent samples *t*-test comparing raw PNT change scores by antidepressant use across both treatments. SE = standard error.

Treatment/ Apraxia of Speech	n	Mean PNT change	SE	df	|t|	Two-tailed *p*-value
**Post-phonological treatment**						
No	39	-1.0	.89	95	3.233	.002
Yes	58	3.8	1.06			
**Post-semantic treatment**						
No	39	3.8	1.15	95	.358	.721
Yes	58	4.5	1.36			

Supplementary Table 11. Independent samples *t*-test comparing raw PNT change scores by presence/absence of apraxia of speech across both treatments. SE = standard error.

Aphasia Type	Phonological raw change (SE)	Semantic raw change (SE)	*t*-statistic	*p*-value
Anomia (n = 25)	1.60 (1.18)	3.28 (1.71)	$t(11) = .714$	.482
Broca's (n = 46)	2.85 (1.34)	4.85 (1.60)	$t(45) = 1.110$	.273
Conduction (n = 16)	.38 (1.91)	5.81 (1.98)	$t(15) = 1.996$	.064
Global (n = 4)	2.00 (1.78)	2.25 (2.93)	$t(3) = .102$	.920
Wernicke's (n = 6)	.500 (.80)	2.58 (.80)	$t(5) = .027$	.027*

Supplementary Table 12. A paired-samples *t*-test comparing the response to phonological and semantic treatment (raw PNT change) for participants grouped by aphasia type. * $p < .05$. SE = standard error.

Variable	Estimate	SE	ß	*t*	R^2 change	Adj. R^2	*p*-value
	--	--	--	--	--	--	--

Supplementary Table 13. Stepwise regression model for post-phonological treatment raw residuals (accounting for the variability explained by the Western Aphasia Battery Aphasia Quotient (WAB-AQ) and post-semantic treatment raw change). Stepping method criteria used probability of F: entry = .05, removal = .10. No variables were included in the final model. SE = standard error.

Variable	Estimate	SE	ß	*t*	R^2 change	Adj. R^2	*p*-value
PALPA 14	-.872	.270	-.586	3.232	.343	.310	.004

Supplementary Table 14. Stepwise regression model for post-semantic treatment raw residuals (accounting for the variability explained by the Western Aphasia Battery Aphasia Quotient (WAB-AQ) and post-phonoogical treatment raw change). Stepping method criteria used probability of F: entry = .05, removal = .10. PALPA = Psycholinguistic Assessment of Language Processing in Aphasia; SE = standard error.

Measure	Treatment: Phonological treatment response	Semantic treatment response	Implications
Phonological processing	(i)	(i) SWR (DV: Sem Resid (Raw), n = 27) identified PALPA 14 score (ß = -.59).<br><br>(ii) High scorers on PALPA 8 (Q4) were more likely to be responders than low scorers (Q1) (n = 44, *p*<.05).	Phonological processing skills may be associated with response semantic treatment, but the nature of the relationship requires further examination.
# of phonological speech errors	--	--	--
Semantic processing	--	--	--
# of semantic speech errors	--	--	--
Apraxia of Speech	(i) Responders (Q4) were more likely to present with apraxia of speech than nonresponders (Q1) (n = 48, *p*<.01).		Presence of apraxia of speech may be associated with response to phonological treatment.
Fluency	--	--	--

Supplementary Table 15. Treatment-specific predictors of raw change colligated across statistical analyses. DV = dependent variable, PALPA = Psycholinguistic Assessment of Language Processing in Aphasia (PALPA 8: nonword repetition; PALPA 14: rhyme judgement requiring picture selection), PPTT = Pyramids and Palm Trees Test, Q = quartile (1: first quartile; 4: fourth quartile), Sem Resid = residuals of semantic proportion of maximum gain regressed on Western Aphasia Battery Aphasia Quotient and phonological proportion of maximum gain; SWR = stepwise regression.

**Measure**	Post-Phon Tx PMG	Post-Sem Tx PMG
Post-Phon Tx raw change	.715**	.018
Post-Sem Tx raw change	.007	.697**

Supplementary Table 16. Pearson‘s correlation coefficients between raw and proportion of maximum gain scores across treatments. Post-Phon Tx = Post-phonological treatment; Post-Sem Tx = Post-semantic treatment. ** denotes statistical significance at $p < .001$.

Supplementary Material

Figures

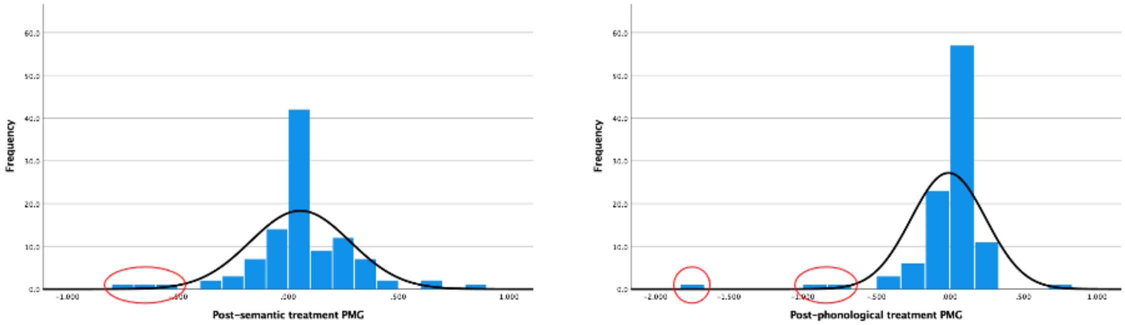

Supplementary Figure 1. Distribution of Outcomes. Histograms showing distribution of the primary outcome measure. Outliers are encircled. PMG = Proportion of Maximal Gain.

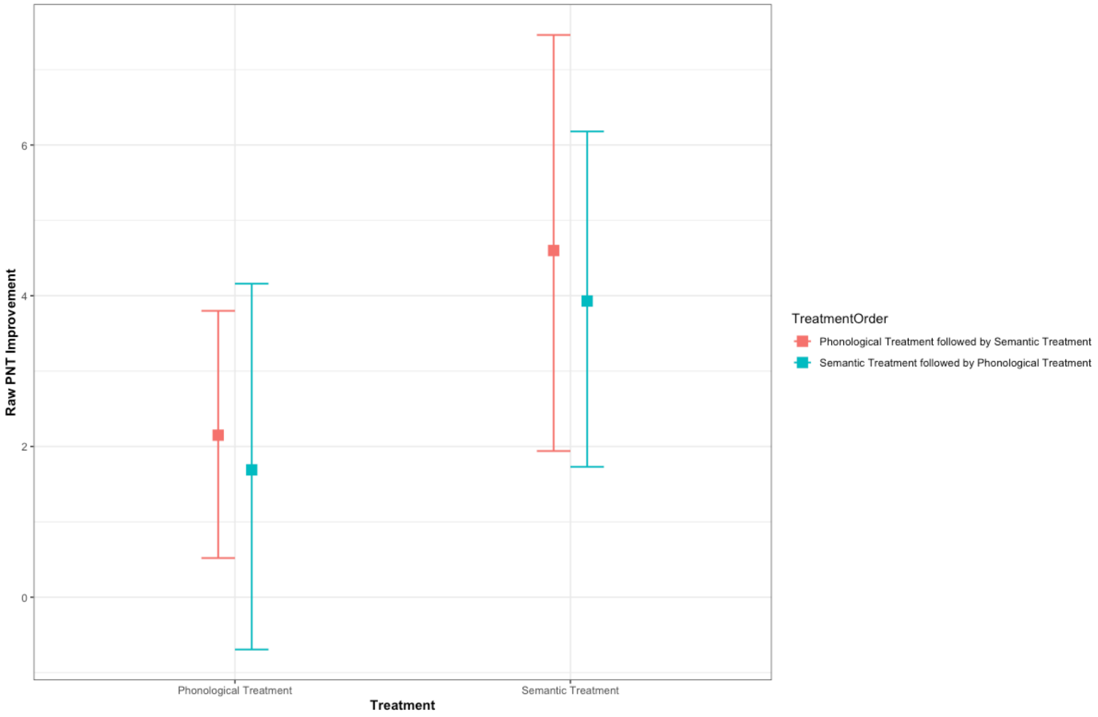

Supplementary Figure 2. Group-Specific Treatment Effects of Raw Scores. Raw PNT change by treatment type across treatment groups (phonological treatment first, n=50; semantic treatment first, n=49). Whiskers denote 95% confident intervals of sample means.

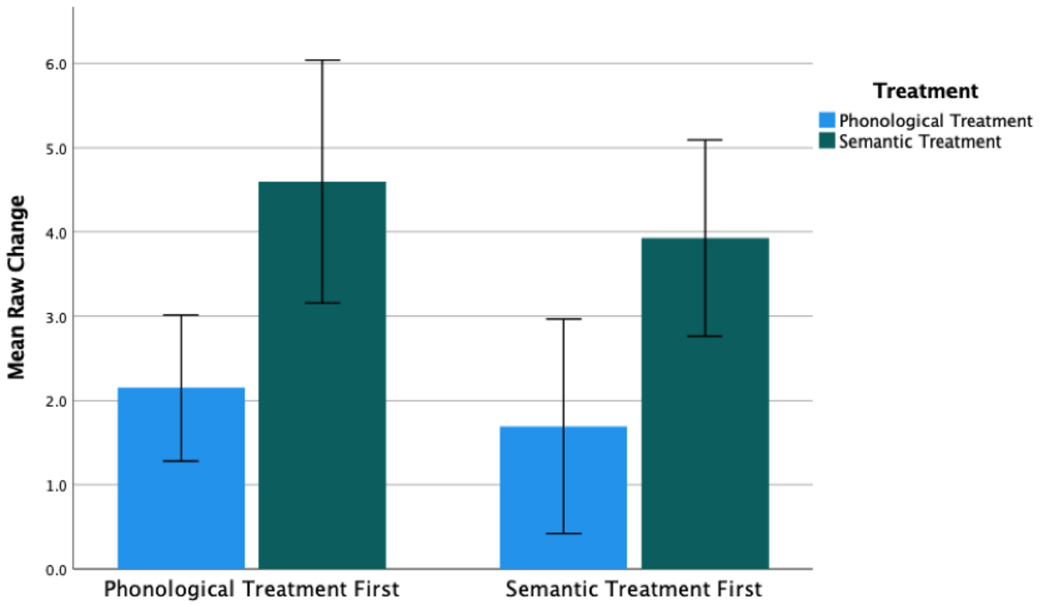

Supplementary Figure 3. Post-Hoc Comparison of Raw Change Across Groups. Mean raw PNT change following phonological and semantic treatment by treatment group (i.e., phonological treatment first, n=50 vs. semantic treatment first, n=49). Whiskers show standard errors of the means.

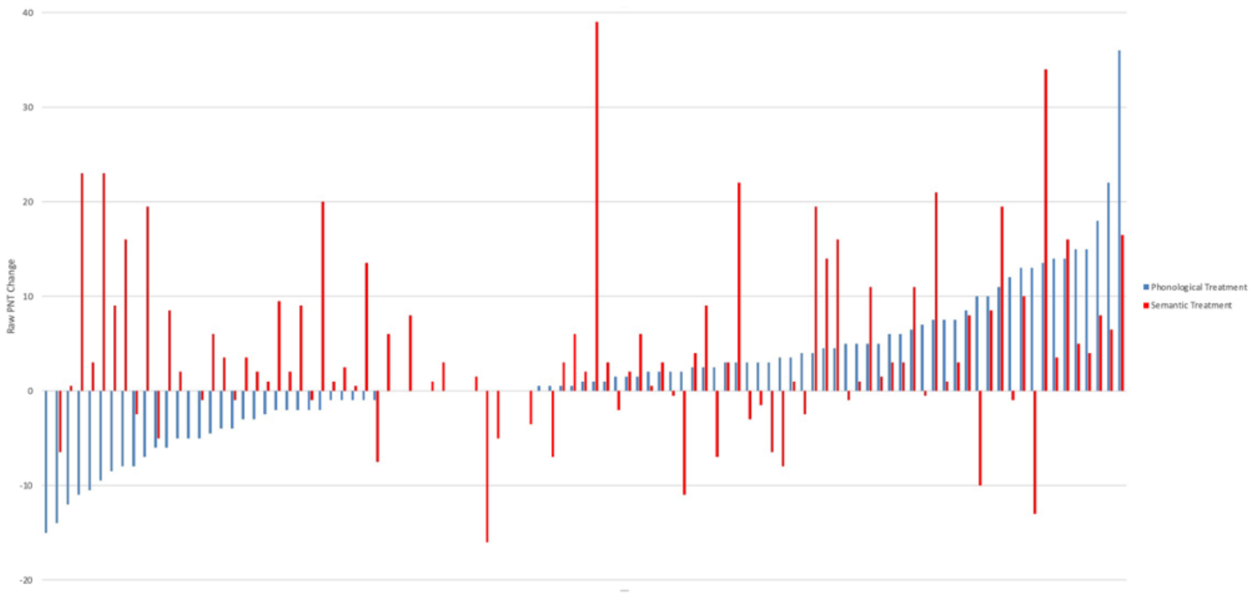

Supplementary Figure 4. Individual Raw Responses to Phonological and Semantic Therapy. Within-individual raw PNG change following phonological and semantic treatment, ordered from the lowest to highest change following phonological treatment and overlaid with the corresponding change following semantic treatment (phonological treatment first, n=50; semantic treatment first, n=49).

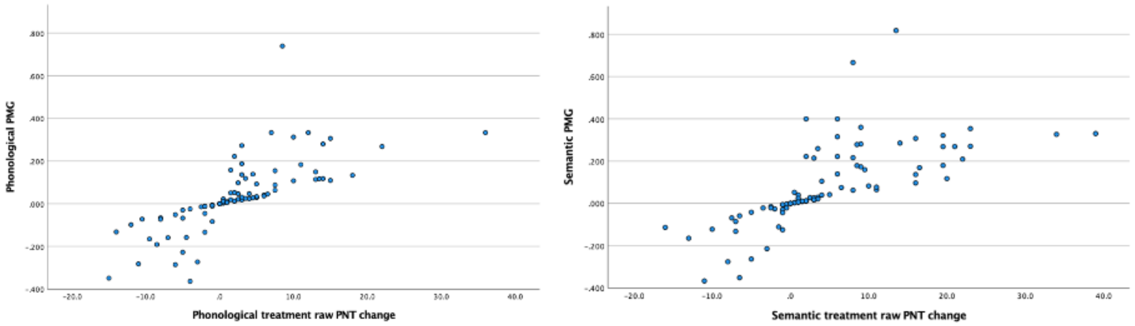

Supplementary Figure 5. Association Between Raw and PMG Values. A scatterplot of raw PNT change score (x-axis) vs. proportion of maximum gain change score (y-axis).

Supplementary References

Bak, T. (2003). Kissing and Dancing—A test to distinguish the lexical and conceptual contributions to noun/verb and action/object dissociation. Preliminary results in patients with frontotemporal dementia. *Journal of Neurolinguistics*, *16*, 169–181. https://doi.org/10.1016/S0911-6044(02)00011-8

Brott, T., Adams, H. P. J., Olinger, C. P., Marler, J. R., Barsan, W. G., Biller, J., Spilker, J., Holleran, R., Eberle, R., & Hertzberg, V. (1989). Measurements of acute cerebral infarction: A clinical examination scale. *Stroke*, *20*(7), 864–870. https://doi.org/10.1161/01.str.20.7.864

Cho-Reyes, S., & Thompson, C. K. (2012). Verb and sentence production and comprehension in aphasia: Northwestern Assessment of Verbs and Sentences (NAVS). *Aphasiology*, *26*(10), 1250–1277. https://doi.org/10.1080/02687038.2012.693584

Grimes, N. (2005). Walt Disney's Cinderella. New York, NT: Random House.

Howard, D., & Patterson, K. (1992). *The Pyramids and Palm Trees Test: A test of semantic access from words and pictures*. Cambridge: Thames Valley Test Company.

Kay, J., Lesser, R., & Coltheart, M. (2009). *PALPA: Psycholinguistic Assessment of Language Processing in Aphasia*. New York, NY: Psychology Press.

Kertesz, A. (2007). *Western Aphasia Battery-Revised*. San Antionio, TX: Pearson.

Lau, M. (2013). Who made that? New York Times Magazine, June 7.

Martin, N., Kohen, F., Kalinyak-Fliszar, M., Soveri, A., & Laine, M. (2012). Effects of working memory load on processing of sounds and meanings of words in aphasia. *Aphasiology*, *26*(3–4), 462–493. https://doi.org/10.1080/02687038.2011.619516

Menn, L., Ramsberger, G., & Estabrooks, N. H. (1994). A linguistic communication measure for aphasic narratives. *Aphasiology*, *8*(4), 343-59.

Roach, A., Schwartz, M., Martin, N., Grewal, R., & Brecher, A. (1996). The Philadelphia Naming Test: Scoring and rationale. *Clin. Aphasiol.*, *24*.

Strand, E. A., Duffy, J. R., Clark, H. M., & Josephs, K. (2014). The Apraxia of Speech Rating Scale: A tool for diagnosis and description of apraxia of speech. *Journal of Communication Disorders*, *51*, 43–50. https://doi.org/10.1016/j.jcomdis.2014.06.008

Wechsler, D. (2008). Wechsler adult intelligence scale–Fourth Edition (WAIS–IV). *San Antonio, TX: NCS Pearson*.
